# *Miscellaneous Remarks on the Small-Pox and Kine-Pock*. *Communicated to* Dr. Mitchill

**Published:** 1804-04-01

**Authors:** Moses Younglove


MISCELLANEOUS REMARKS ON THE SMALL-POX AND KINE-POCK. *Communicated to* DR. MITCHILL *by* DR. MOSES YOUNGLOVE, *in a Letter dated New Lebanon Springs, February* 12, 1803.

CONSIDERING the hopeful progress of vaccination, any communication and discussion relative to the *small-pox* may, to some, seem unseasonable; yet, notwithstanding the flattering flattering prospect that the former will generally supersede the latter, as to the practice of inoculation, in all lettered States, at a time not very far distant, the prevalence of the latter, for reasons well known, is still considerable; and since, through its very infectious nature, and the great intercourse by commerce, this may, for a time, remain the case; and since investigations on any disease are more or less favourable to the study of every kindred disease, I take the freedom of offering to your inspection a few desultory remarks, with reference to what hath appeared in several publications relative to the small-pox.

A question is controverted by pathologists, whether two or more contagious diseases can operate at once on the same subject. It appears by the last number of the Medical Repository, that Dr. J. R. Coxe, in his late treatise on vaccination, which I regret that I have not seen, advocates the affirmative of this question; while Mr. J. Hunter, Dr. E. Darwin, and several others, maintain the contrary; and some of them make of that opinion a theoretic basis to very important deductions. From long and diligent observation, I am fully confirmed in Dr. Coxe's opinion in the premises. Many instances have occurred of my patients having, at the same time, the small-pox and measles, the small-pox and chicken-pox, the small-pox and the venereal disease, the small-pox and the mumps, and the small-pox and hooping-cough; and, as far as I could see, both diseases, in each case, proceeded in their usual progress, without any suspension of the operation of the one by the other, as several writers have supposed. Nor did the two diseases, that I know of, coalesce, and form a third intermediate *something of a disease*, as Dr. Darwin ludicrously supposes, (Zoon. §. 33. 2, 9) though they, in several cases, appeared to be in even progress; and in one instance, about the year 1785, I had a patient, when the measles were prevalent, who, about the thirteenth day after inoculation, and after pretty severe symptoms, broke out with the small-pox and measles both at once; but the latter appeared entirely apart, in blotches of an inch or two diameter; after which both happily proceeded in their natural operation.

As to the opinion espoused by Dr. Coxe, and other reputable authors, that some persons have had the small-pox more than once, as I have no proof to the contrary, so neither have I, in my practice, seen any evidence in favour of it. I once, through mistake, inoculated a family with infection of the chicken-pox instead of the small-pox, when when the operation produced, though slight, yet did not undeceive me (I being then young in the practice), till the person out of whom I had taken the infection, happened, under my eye, about a year after, to break out with the natural small-pox, which admonished me to inoculate them anew with the true infection; but had it not been for this seasonable discovery, these patients might, many years after, have had the small-pox by the natural way, and I might have confidently asserted that they had the disease a second time.

I have also known several instances in which practitioners of little experience have inoculated for the small-pox, with matter adulterated with the itch, or the like, which producing a sore, and a kind of eruption, were pronounced safely through the intended disease, yet have afterwards caught it, and then been said, as confidently, to have it a second time.

It is also asserted, by some writers, that there are, in every country, some to whom the small-pox cannot be given by inoculation, although they have never had it before trial; but in the course of my experience during the revolutionary war of these States, and since, on, I believe, not less than thirteen or fourteen thousand subjects, I have found none such when my infection was good; though I know several persons who, having had the disease very slightly, are generally thought to be of that description.

Nor have I ever, but once, seen the operation of the spurious or imperfect small-pox mentioned by Dr. Coxe sometimes to occur, which was about thirteen years since, when I began inoculation, between Kinderhook and Claverack, with old infection, procured from a neighbouring physician; but very singular was its operation; its taking effect was generally uncertain without repeated inoculations; its whole subsequent operation was mostly so slight, and often partial too, producing only a scabby eruption on the inoculated arm, that my utmost efforts could not, in every instance, produce an operation fully satisfactory, though a very few had the disease severely, with a natural appearance enough. This continuing the case for several weeks, while I could procure no other infection, but inoculated every succeeding class and family with the best I could select from the preceding, I was constrained, merely on that account, to desist, though reluctantly, after having inoculated nearly two hundred.

There is a query (Med. Rep. vol. i. p. 103) whether, in inoculation with the small-pox, the insertion of an unnecessary cessary quantity of the matter will increase the virulence of the disease? To this I answer, that though I have never plainly perceived any injury from this source on strong subjects, yet on tender infants, and weak patients in general, I have thought the insertion of much, especially by deep incision, and more especially if repeated in several places, to be very injurious, apparently through the unnecessary corrosion of the skin, cellular membrane, and even muscular flesh, and the consequent fever, depression, and the early and unnatural absorption of the matter into the mouths of the lymphatics.

As to the advantage there mentioned from a dilution of the virus before ingrafting, I am fully of opinion that it does in no case tend to the proposed mitigation of the disease, but that it is highly improper, rendering the communication of the disease far less certain, if a mixture of water, or any other extraneous matter, be unnecessarily admitted. And hence the reason is evident why the first limpid matter in the pustules is so very sure to communicate the disease by inoculation; this young matter being naturally the most pure from any mixture of common pus, or other foreign material.

Dr. Darwin queries (Zoon. § 33. 3, 1), whether the infection of the small-pox can be effectually cut out again after inoculation. I can partly answer this, by stating that, about fifteen years since, on the alarming illness of one of my patients (at Lunenburg, near the city of Hudson), with the appearance of an incipient yellow fever, on the third day after an inoculation which had undoubtedly taken effect, I freed him from it by cutting out a piece of skin less than a dime; but that I have since failed in a similar attempt, the fourth day after inoculation, in a manner, however, indicating, that if my incision had been made wider and deeper it might have succeeded: for about the ninth day after inoculation, as in other cases, the sore was surrounded with the true pock inflammation, and the eruptive progress following was as usual.

Dr. Darwin (Zoon. § 33. 2, 10) acknowledges his recent conviction, that the variolous matter never circulates in the blood of any person infected with it, as evinced by several experiments of inoculating with the blood without effect. Yet, notwithstanding this rational conviction of him and others, the position is controverted, and, as far as I am acquainted, generally discredited. As for myself, I was more than twenty years ago conviced of its truth, and have since been more and more confirmed in this conviction, tion, by having observed the following among other evidences of it.

1. Generally about five or six days from the inoculation all its observable operation is confined to the infected spot, or very near it; for a few days longer its progress from that is mostly slow and gradual; and then it first affects the glands and muscles on the same side, about the shoulder, neck, and head.

2. The following progress of the eruptive symptoms is very similar to that of various other potent irritations.

3. I have inoculated, I believe, much more than a hundred women, in the early and middle stages of pregnancy, with safety in most cases; and have frequently, long afterward, inoculated the children so born of them, who have invariably undergone the usual operation of the disease, which might not have been the case had the variolous matter pervaded them in utero.

Nor hath the argument any weight with me, when some former writers urge the infectious nature of the pustules emanating from the blood, as a proof of the presence of variolous matter in that fluid; for I have diligently sought, in vain, for any evidence of infection in the incipient pustules before maturation; and they never maturate but where they are exposed to the air; a portion of which may, perhaps, for that end, form a chemical union with some of the secretions of the patient, essential to their contagious maturity; and hence, in abortions from this disease, in the last stage of gestation, the pimples frequently observable on the fœtus have none of the variolous matter in them.

4. I have never known any person take the small-pox from the blood, though exposed to it, fresh drawn, at the most likely season, aftor the absorption into the lymphatics of a profuse and crystalline eruption, which is sometimes astonishing, both as to the quantity of matter receding from sight, and the quickness of its recession: insomuch, that I have once seen an instance where the bladdery cap of a large, full, remaining cyrstalline pock, was hastily pulled off, with a view, by applying a sponge, to hinder the retreat of its contents into the system, when it instantly ran in under my eye, before the sponge could be properly applied, and that too on the under-side of the patient's leg, in opposition to centripetal attraction. Yet, in some of these cases, when induced by improper treatment, on a seasonable resort to a warm and cordial regimen, and to the proper internal remedy, one may the next day see the happy return of the matter in large blisters, at or very near the pustules it before filled (though now so far divested of its late keen contagious virulence as to be very uncertain to inoculate with). It therefore appears to me probable, that its retrogression was only into the mouths of the lymphatics, which naturally inflames them, indurates their glands, and induces dangerous fevers, by the associate and sympathetic motions of the different parts of the system.

The writings of some transatlantic authors, formerly of credit, would induce a belief that it was once thought there, that the small-pox was not only modified in operation by particular sites and seasons of the year, but was frequently originated by these causes. These notions appear as the mere reveries of inexperience and error: but many descriptions of the disease, its operation and proper management, by writers of the best credit, such as Huxham's little black pock, which never was seen here in our day, &c. and many observations by Sydenham and others, his cotemporaries, equally variant from our experience, may perhaps be credited as then correct, by supposing a general change in the operation of the disease, gradually down the lapse of time from them to us; which is to me the more probable, because I think I can distinctly trace, by memory, a transition of this kind since I began to practice in the disease, which is about twenty-eight years. I see no more of the glandular suppurations in the latter part of the disorder, formerly so frequent and unavoidable. I can say almost the same of the rash, an eruption so common formerly at the close of the eruptive illness. Also the scarlet, or sometimes crimson efflorescence, well described by the acute Dr. Brown (Elem. Med. p. 219 and 421); also a border of like appearance round the inoculated spot on tender infants, near the time of eruption, varying in different shades of delicate colours to the eye, in transient and momentary flushes. The two last I have not seen these ten or twelve years, though formerly of pretty frequent occurrence in my practice. And, in general, I think it evident that the small-pox here hath grown far more mild and manageable than formerly under similar treatment.

